# Neural Correlates of Central Inhibition during Physical Fatigue

**DOI:** 10.1371/journal.pone.0070949

**Published:** 2013-07-26

**Authors:** Masaaki Tanaka, Akira Ishii, Yasuyoshi Watanabe

**Affiliations:** 1 Department of Physiology, Osaka City University Graduate School of Medicine, Osaka City, Osaka, Japan; 2 RIKEN Center for Life Science Technologies, Kobe City, Hyogo, Japan; Tokyo Metropolitan Institute of Medical Science, Japan

## Abstract

Central inhibition plays a pivotal role in determining physical performance during physical fatigue. Classical conditioning of central inhibition is believed to be associated with the pathophysiology of chronic fatigue. We tried to determine whether classical conditioning of central inhibition can really occur and to clarify the neural mechanisms of central inhibition related to classical conditioning during physical fatigue using magnetoencephalography (MEG). Eight right-handed volunteers participated in this study. We used metronome sounds as conditioned stimuli and maximum handgrip trials as unconditioned stimuli to cause central inhibition. Participants underwent MEG recording during imagery of maximum grips of the right hand guided by metronome sounds for 10 min. Thereafter, fatigue-inducing maximum handgrip trials were performed for 10 min; the metronome sounds were started 5 min after the beginning of the handgrip trials. The next day, neural activities during imagery of maximum grips of the right hand guided by metronome sounds were measured for 10 min. Levels of fatigue sensation and sympathetic nerve activity on the second day were significantly higher relative to those of the first day. Equivalent current dipoles (ECDs) in the posterior cingulated cortex (PCC), with latencies of approximately 460 ms, were observed in all the participants on the second day, although ECDs were not identified in any of the participants on the first day. We demonstrated that classical conditioning of central inhibition can occur and that the PCC is involved in the neural substrates of central inhibition related to classical conditioning during physical fatigue.

## Introduction

Fatigue, defined as difficulty in initiating or sustaining voluntary activities [1], can be classified as physical or mental, and physical fatigue can be classified as peripheral or central. During a physical task, the primary motor cortex (M1) is activated and this leads to the excitation of motoneurons in the spinal cord. The axon of the motoneuron in the spinal cord conveys action potentials to the neuromuscular junction of the muscle. The peripheral nerve, neuromuscular junction, and muscle are referred to as peripheral factors. Muscle fatigue is defined as a progressive decline in maximal voluntary force produced by a muscle or muscle group [2]. Accumulation of intracellular lactate and hydrogen ions has not been associated with muscle fatigue [3], [4]. Alternatively, depletion of glycogen, ATP, and phosphocreatine, effects of ionic changes on the action potential, failure of sarcoplasmic reticulum Ca^2+^ release, and effects of reactive oxygen species are considered to be associated with muscle fatigue [5].

In contrast to the peripheral fatigue, central fatigue is caused at sites proximal to the peripheral nerve and is defined as a progressive decline in the ability to activate muscles voluntarily [6], [7]. Increased inhibition from groups III and IV afferent nerves, which carry sensory information to the central nervous system, to motor neurons in the spinal cord, was reported during physical fatigue [8]–[12]. The inhibitory input to the motoneuron pool in the spinal cord projects to output neurons in M1 through ascending pathways and the inhibitory input changes the signals of these cells in M1 [13]. The inhibitory input from the spinal cord thus seems to contribute to fatigue-related changes in M1, although the supraspinal mechanisms are unclear.

A Ramachandran's mirror box was constructed by placing a vertical mirror inside a cardboard box with the roof of the box removed; the front of the box had two holes in it through which the participants inserted their arms [14]. Recently, using this mirror box, we found that reduced left handgrip force caused by performing repetitive left handgrips at maximal voluntary contraction levels was attenuated by looking at the mirror reflection of the right hand to perceive that the left hand was not fatigued [15]. This finding suggests (1) that the supraspinal existence of central inhibition to M1 via mirror visual feedback during physical fatigue, (2) that the visual feedback system is involved in the mechanisms regulating motor output and thus physical performance, and (3) that feedback conflicting with an individual motor control, plan, realization, or sensory input is adjusted through this system. In addition to our behavioral study, the existence of central inhibition to M1 during physical fatigue was suggested in a neuroimaging study using the Ramachandran's mirror box [16]: Although the movement-evoked magnetoencephalography (MEG) response to the imagery of maximum voluntary contractions in the contralateral sensorimotor area was reduced by physical fatigue without the mirror box, the reduction was completely disappeared with the mirror box. These results confirmed that sensory input was channeled to the ipsilateral M1 using the mirror box, and provided evidence for supraspinal existence of central inhibition during physical fatigue [16]. Taking into consideration that behavioral data showed that the increased inhibitory input was responsible for 20 to 25% of the force loss due to physical fatigue [15], and that central inhibition was shown to be responsible for 20 to 25% of the force loss due to physical fatigue [17], it seems likely that central inhibition primarily contributes to central or supraspinal fatigue [18].

Central inhibition plays an important role as a biological alarm under the conditions of physical fatigue [18] as well as mental fatigue [19], and thus urges us to take a rest to avoid upsetting homeostasis and to recover from fatigue. In this sense, central inhibition is beneficial for our survival. However, enhanced activation of central inhibition seems to be involved in the pathophysiology of fatigue-related diseases, such as chronic fatigue syndrome, an illness characterized by a profound, disabling, and unexplained sensation of fatigue lasting at least 6 months, which severely impairs daily functioning and is accompanied by a combination of nonspecific symptoms [20]. One hypothesis, named the “Co-conditioning Theory of Chronic Fatigue,” was proposed as an etiology of chronic fatigue; this hypothesis supposes that enhanced activation and classical conditioning of central inhibition as a result of repeated fatigue assaults are associated with the pathophysiology of chronic fatigue syndrome [21]. Therefore, it is important to clarify the mechanisms of central inhibition as well as the mechanisms involved in the conditioning of central inhibition.

Recently, we performed a neuroimaging study of classical conditioning of mental fatigue [22]. In this study, metronome sounds were used as conditioned stimuli and fatigue-inducing mental task trials were used as unconditioned stimuli to cause mental fatigue. Participants underwent MEG measurements while listening to the metronome sounds for 6 min. Thereafter, fatigue-inducing mental task trials were performed for 60 min; metronome sounds were started 30 min after the beginning of the task trials. The next day, neural activities during listening to the same metronome sounds for 6 min were measured. The level of fatigue sensation caused by listening to the metronome sounds on the second day was increased relative to the first day and the equivalent current dipoles (ECDs) in the insular cortex and posterior cingulate cortex (PCC) were observed only after the conditioning session. These results showed that classical conditioning of mental fatigue did take place and that these brain regions were involved in the neural substrates of mental fatigue, or rather, mental fatigue sensation related to classical conditioning. However, it was not clear whether classical conditioning of central inhibition occurred during physical fatigue. In addition, it was not clear whether these brain regions were involved in the neural substrates of central inhibition related to classical conditioning during physical fatigue.

The aims of the present study were therefore to determine whether classical conditioning of central inhibition can occur and to identify the neural substrates of central inhibition related to classical conditioning during physical fatigue. Similar to our previous study [22], we tried to get classically conditioned participants to experience central inhibition when they were listening to sounds, using fatigue-inducing physical task trials as unconditioned stimuli and metronome sounds as conditioned stimuli. We compared neural activities between the unconditioned and conditioned states. Because high-resolution temporal sequences may provide important clues to clarify the neural mechanisms of classical conditioning of central inhibition [22], we used MEG to assess neural activities.

## Methods

### Participants

Eight healthy male volunteers (age, 29.3±10.2 years [mean±SD]) were enrolled. According to the Edinburgh handedness inventory [23], all the participants were right-handed. Current smokers, participants with a history of mental or brain disorders, and those taking chronic medications that affect the central nervous system were excluded. All the participants provided written informed consent before participation. This study was approved by the Ethics Committee of Osaka City University and was conducted in accordance with the principles of the Declaration of Helsinki.

### Experimental design

**Figure 1:**
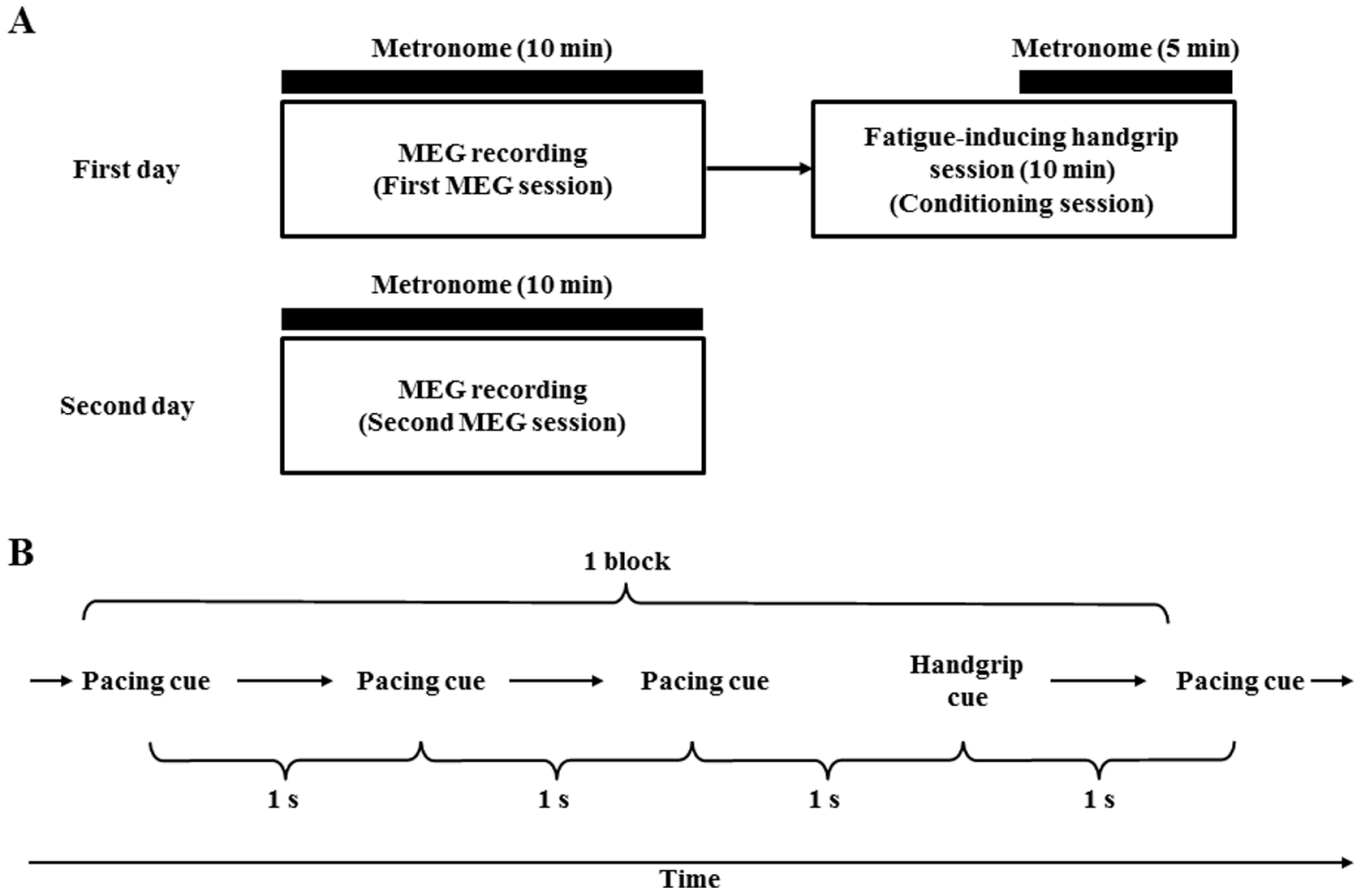
Experimental paradigm. (A) Experimental design. On the first day, neural activities during the imagery of handgrips guided by the handgrip cues of metronome sounds were measured using magnetoencephalography (MEG) for 10 min (First MEG session). Thereafter, 10-min fatigue-inducing maximum handgrip trials (Conditioning session) were performed, in which metronome sounds were started 5 min after the beginning of the handgrip trials as conditioned stimuli. On the next day, neural activities during the imagery of handgrips guided by the handgrip cues of metronome sounds were measured using MEG for 10 min (Second MEG session). (B) Metronome sounds. Each MEG session consisted of 150 blocks, and each block consisted of three pacing cues followed by one handgrip cue. During the MEG session, participants heard the sound cues every 1 s with their eyes closed, and during the handgrip cue period, they were requested to imagine that they were gripping a soft ball with their right hand at a maximal voluntary contraction level every 4 s for 1 s.

The experiment consisted of two MEG sessions and a single conditioning session (Fig. 1A). On the first day, MEG recording during imagery of maximum grips of the right hand, guided by metronome sounds, was performed for 10 min (first MEG session). Thereafter, 10-min fatigue-inducing maximum handgrip trials using a device (HAND GRIPS 30 kg; IGNIO, Nagoya, Japan) were performed (conditioning session), in which the metronome sounds (same as the MEG session) were started 5 min after the beginning of the handgrip trials and the sounds were continued until the end of the handgrip trials as conditioned stimuli [22]. The participants were not informed about the metronome sounds before the task trials. On the next day, MEG recording during imagery of maximum grips of the right hand guided by metronome sounds was performed for 10 min (second MEG session).

Each MEG session consisted of 150 blocks, and each block consisted of three pacing cues followed by one handgrip cue (Fig. 1B). During the MEG session, the participants heard the sound cues every 1 s with their eyes closed, and every 4 s during the handgrip cue period, they were requested to imagine that they were gripping a soft ball with their right hand at a maximal voluntary contraction level for 1 s. The pacing cue consisted of white noise that lasted 33 ms; the handgrip cue consisted of a 1000 Hz tone that lasted 1 s. All the cue sounds were produced by Windows Media Player (Microsoft Corporation, Redmond, WA) and were converted to electric signals by a sound card (Creative X-Fi Audio Processor [WDM]; Creative Technology, Singapore, Singapore) installed in a personal computer (DELL Precision 390; Dell, Round Rock, TX). The sound signal was amplified by an audio amplifier (MA-500U; Onkyo Corporation, Tokyo, Japan) outside of the magnetically shielded room.

During the conditioning session, they watched a fixed mark (+; black mark on white background) on a screen placed in front of their eyes using a video projector (PG-B10S; SHARP, Osaka, Japan). When a handgrip cue mark (×; black mark on white background) was presented instead of the fixation mark every 4 s, they were requested to perform a handgrip with their right hand at a maximal voluntary contraction level for 1 s by gripping the device. The timing of the visual handgrip cues was same as that of the metronome handgrip cue sounds started 5 min after the beginning of the handgrip trials.

Electrocardiography (ECG) was recorded during the MEG sessions. Just before and after the conditioning session and after each MEG session, the participants were asked to subjectively rate their fatigued level of the right and left hands on a visual analogue scale (VAS) from 0 (minimum) to 100 (maximum) [24]. In addition, just before and after the conditioning session, handgrip forces were measured using a handgrip meter (ST-100, Toei Light Co., Ltd, Saitama, Japan).

This study was conducted in a quiet, temperature-, and humidity-controlled, magnetically shielded room. During the experiment, they lay on a bed in a supine position. For 1 day before each visit, they refrained from intense physical and mental activities and caffeinated beverages, consumed a normal diet, and maintained normal sleeping hours.

### MEG recordings

MEG recordings were performed using a 160-channel whole-head type MEG system (MEG vision; Yokogawa Electric Corporation, Tokyo, Japan) with a magnetic field resolution of 4 fT/Hz^1/2^ in the white-noise region. The sensor and reference coils were gradiometers 15.5 mm in diameter and 50 mm at baseline, and each pair of sensor coils was separated at a distance of 23 mm. The sampling rate was 1000 Hz with a 200 Hz hard low-pass filter and a 0.3 Hz hard high-pass filter.

### MEG data analyses

Before processing MEG data, magnetic noise originating from outside the shield room was eliminated by subtracting the data obtained from reference coils using a software program (MEG 160; Yokogawa Electric Corporation). The MEG signal data were averaged offline after analogue-to-digital conversion with a band-pass filter of 3 to 30 Hz. The mean magnetic signal in the pre-stimulus time period (from 0 to 500 ms before the start of each visual stimulus) was subtracted in each channel to remove the baseline shift of MEG data. Epochs of raw MEG data, including artifacts such as eye blinks and eye movements, were excluded from the analysis by careful visual detection of artifacts before averaging. To identify sources of evoked activities, ECD analyses were performed using MEG 160. Latencies and intensities were estimated using ECD analyses. The dipole assignment required a goodness of fit (GOF) value above 85%, based on a previous report [25].

### Magnetic resonance imaging overlay

Anatomical magnetic resonance imaging (MRI) was performed using a Philips Achieva 3.0TX (Royal Philips Electronics, Eindhoven, The Netherlands) for all the participants to permit registration of magnetic source locations with their respective anatomical locations. Before MRI scanning, five adhesive markers (Medtronic Surgical Navigation Technologies Inc., Broomfield, CO) were attached to the skin of each participant's head (the first and second ones were located 10 mm in front of the left tragus and right tragus, the third at 35 mm above the nasion, and the fourth and fifth at 40 mm right and left of the third one). MEG data were superimposed on MRI scans using information obtained from these markers and MEG localization coils.

### ECG

To evaluate autonomic nerve activities, ECG was recorded during the MEG sessions. ECG data were analyzed using MemCalc for Windows (Global Medical Solution Inc., Tokyo, Japan). R-R wave variability was measured as an indicator of autonomic nerve activity. For frequency domain analyses of the R-R wave intervals, low-frequency (LF) power was calculated as the power within the frequency range of 0.04 to 0.15 Hz, and high-frequency (HF) power was calculated as that within the frequency range of 0.15 to 0.4 Hz. LF and HF were measured in absolute units (ms^2^). The average power densities within these frequency ranges were log-transformed (ln) for normalization [26]. The HF is vagally mediated [27]–[29], but the LF originates from a variety of sympathetic and vagal mechanisms [27], [30]. The LF/HF ratio represents sympathetic nerve activity [31].

### Statistical analyses

Values are presented as mean±SD, unless otherwise stated. Two-way analysis of variance (ANOVA) for repeated measures was performed to assess the effects of the hand (right or left) and time course within the conditioning session on subjective level of fatigue. Paired t-test was used to evaluate significant differences between two conditions. Categorical variables were compared using McNemar's test. All P values were two-tailed, and values less than 0.05 were considered statistically significant. Statistical analyses were performed using IBM SPSS 20.0 (IBM, Armonk, NY).

## Results

**Figure 2:**
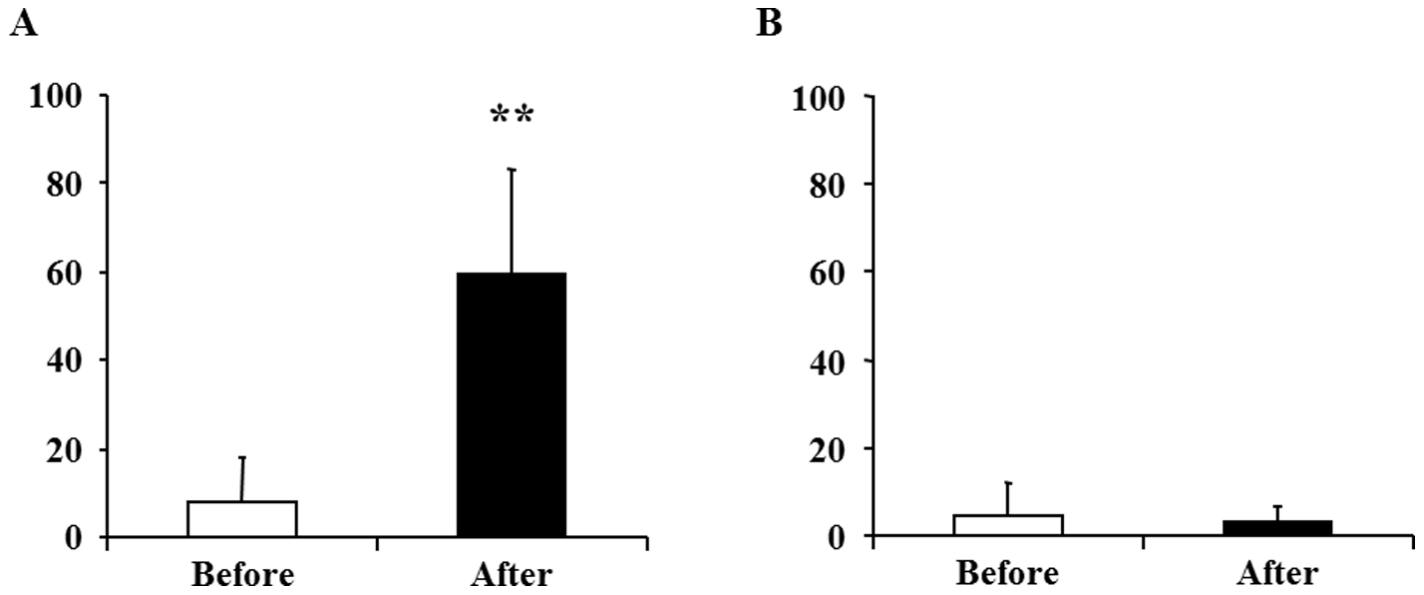
Visual analogue scale (VAS) value of the right (A) and left (B) hands for fatigue immediately before (open columns) and after (closed columns) the 10-min fatigue-inducing handgrip trials. Data are presented as mean and SD. **P<0.01, significantly different from the corresponding values before handgrip trials (paired t-test).

To assess changes in the subjective level of fatigue after the 10-min maximum handgrip trials, two-way ANOVA for repeated measures was performed. Significant main effects of hand [F(1,7) = 47.61, P<0.001] and time course [F(1,7) = 33.99, P = 0.001] and a hand × time course interaction effect [F(1,7) = 32.61, P = 0.001] were shown. The level of subjective fatigue of the right hand after handgrip trials was significantly higher than that before handgrip trials (Fig. 2A). However, the level of subjective fatigue of the left hand was not altered after handgrip trials (Fig. 2B). The handgrip force of the right hand after handgrip trials was significantly lower than that before the trials (before, 39.6±8.5 kg; after, 31.1±9.7 kg; P<0.001, paired t-test).

**Figure 3:**
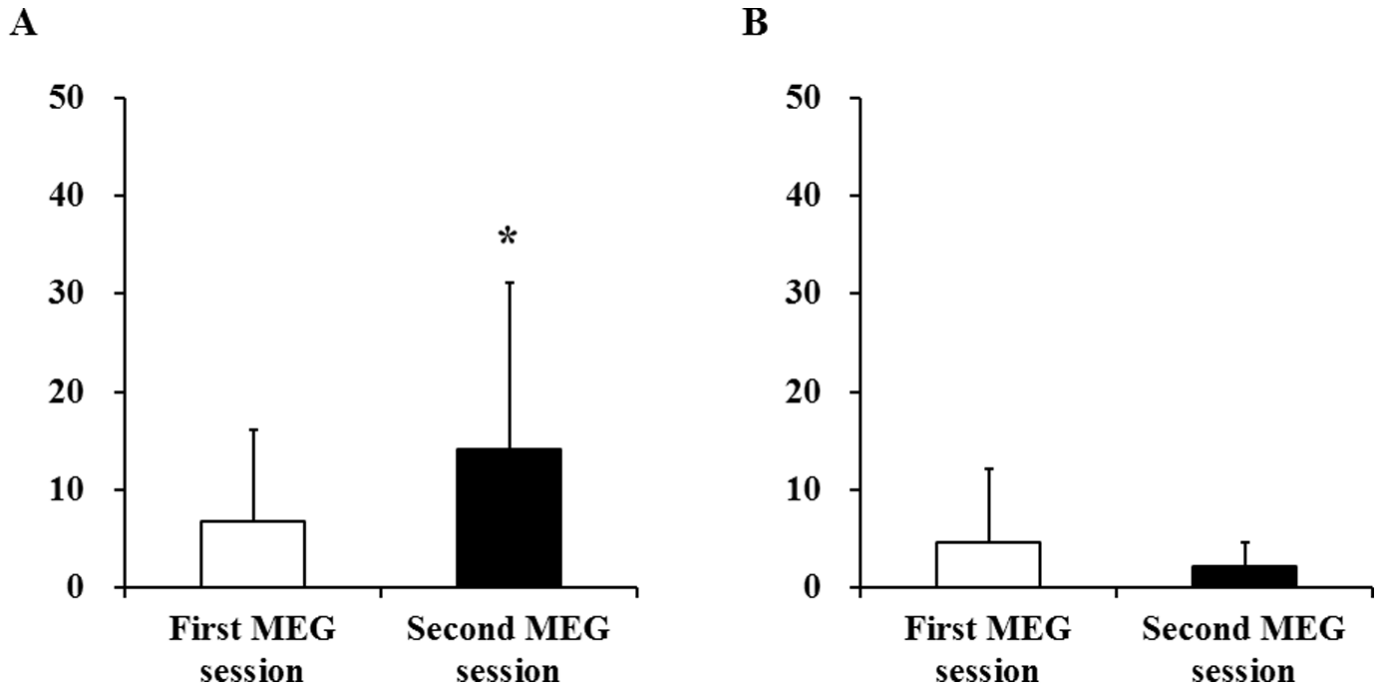
Visual analogue scale (VAS) values of right (A) and left (B) hands for fatigue immediately after the first and second magnetoencephalography (MEG) sessions. Data are presented as mean and SD. *P<0.05, significantly different from the corresponding values of the first MEG session (paired t-test).

To assess changes in the subjective level of fatigue after the conditioning session, comparisons of fatigue scores after the first and second MEG sessions were performed. The subjective level of right hand fatigue after the second MEG session was higher than that after the first MEG session (Fig. 3A), although the subjective level of left hand fatigue after the second MEG session was not different from that after the first MEG session (Fig. 3B).

**Figure 4:**
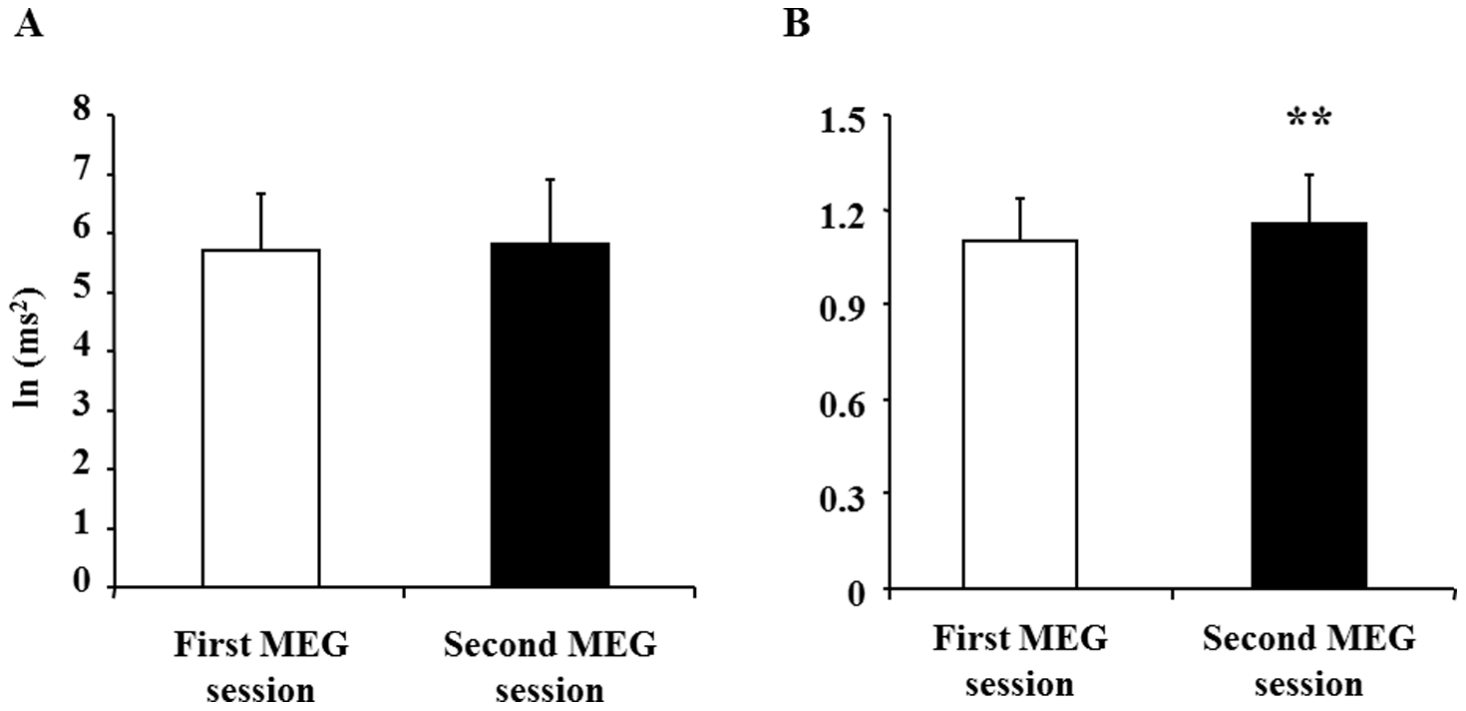
Autonomic nerve activities assessed by frequency analyses of electrocardiography (ECG). High-frequency power (HF; A) and low-frequency power (LF)/HF ratio (B) were assessed during the first and second magnetoencephalography (MEG) sessions. Data are presented as means and SD. **P<0.01, significantly different from the corresponding values of the first MEG session (paired t-test).

We also assessed changes in HF and LF/HF ratio after the conditioning session. Although HF was not altered after the conditioning session (Fig. 4A), LF/HF ratio was significantly increased after the conditioning session (Fig. 4B).

**Figure 5:**
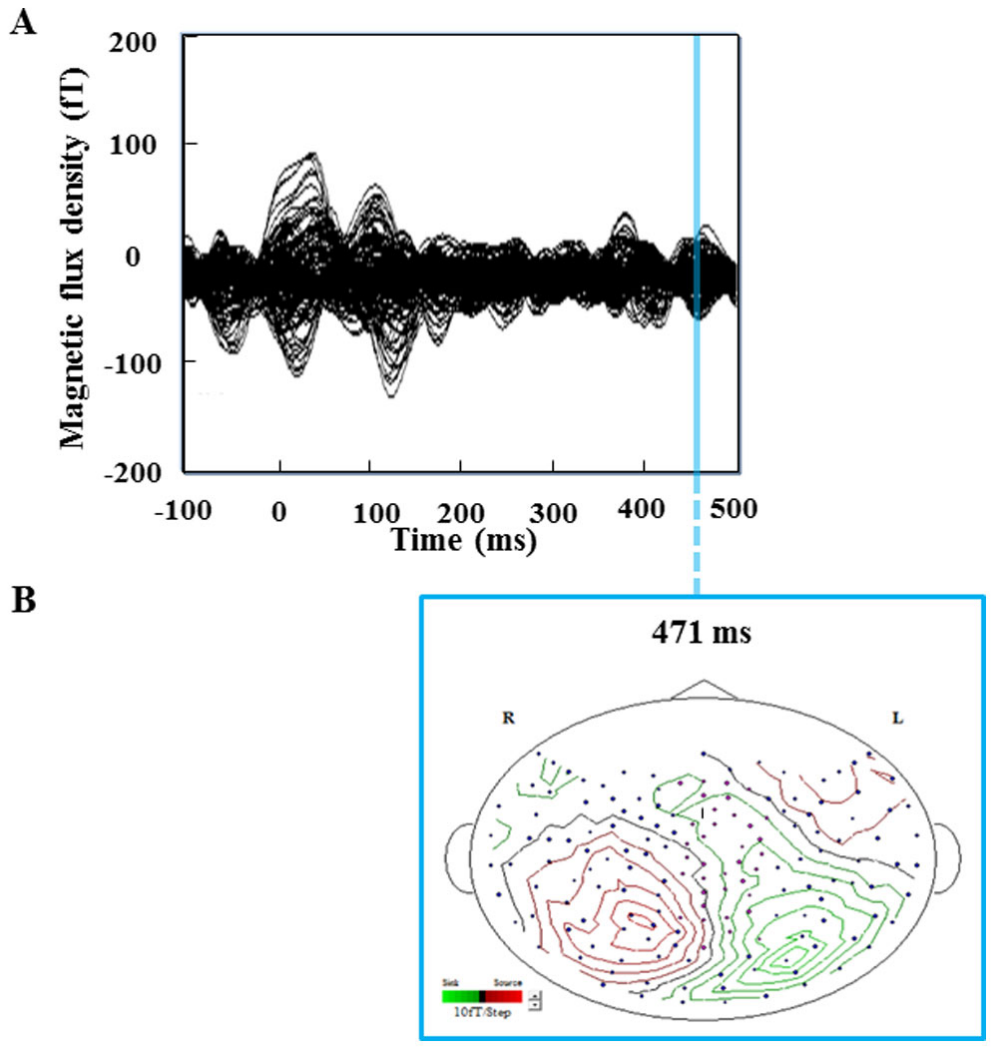
Typical example of magnetic fields (A) and an isofield contour map (B) caused by the cue sounds and imagery of handgrips (participant No. 5). The equivalent current dipole (ECD) in the posterior cingulate cortex is shown at the peak latency of 471 ms. R: right side; L: left side.

A typical example (participant No. 5) of magnetic fields observed in the second MEG session is shown in Fig. 5A. There was one major magnetic response observed only in the second MEG session. The peak latency of the magnetic response after the start of each metronome sound was 471 ms. Isofield contour map corresponding to this magnetic response is shown in Fig. 5B.

**Figure 6:**
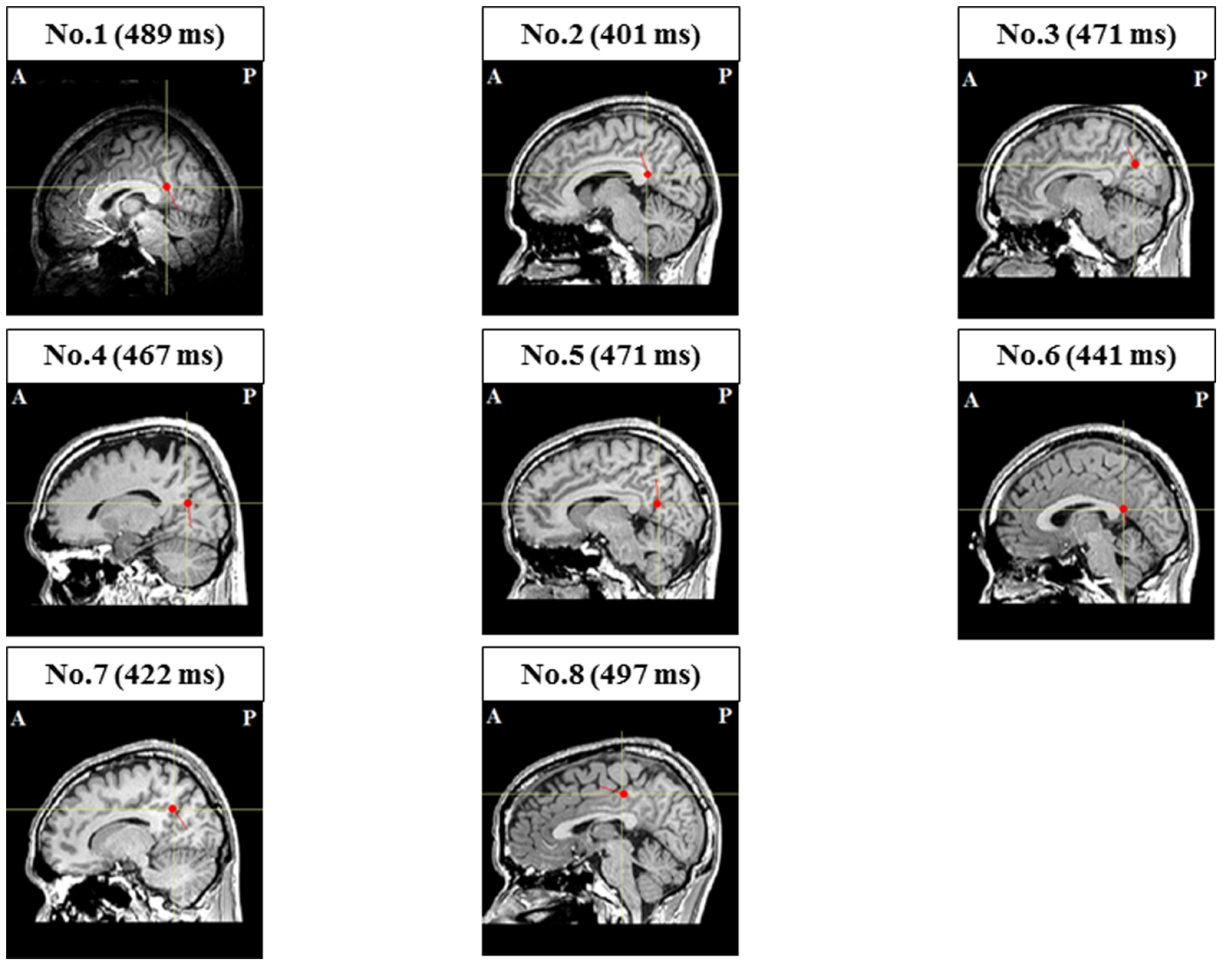
Locations of equivalent current dipoles (ECDs) in the posterior cingulate cortex induced by cue sounds and imagery of handgrips during the second magnetoencephalography (MEG) sessions. Closed red circles indicate the locations of ECDs and short red lines radiating from closed circles indicate the orientation of ECDs. ECDs are superimposed on individual magnetic resonance images (sagittal view) (A: anterior side; P: posterior side). The peak latencies are presented in parentheses.

In the ECD analyses, we could not identify the magnetic responses localized in the PCC in any of participant during the first MEG session, while we could estimate the magnetic responses localized in the PCC in all eight participants during the second MEG session; mean latencies were approximately 460 ms (Table 1 and Fig. 6). The proportion of participants in whom ECDs in the PCC could be estimated in the second MEG session was statistically significant relative to that in the first MEG session (P = 0.008, McNemar's test).

**Table 1:** Properties of the equivalent current dipoles (ECDs) in the posterior cingulate cortex (PCC) during the second magnetoencephalography (MEG) session.

No. of participants	8
Peak latency (ms)	457.4±33.2
GOF (%)	93.8±3.6
Intensity (nAm)	25.9±14.1

Data are presented as mean±SD.

GOF, goodness of fit.

## Discussion

We tried to determine whether classical conditioning of central inhibition can occur and to clarify the neural substrates of central inhibition related to classical conditioning during physical fatigue using MEG. We found that levels of fatigue sensation and sympathetic nerve activity were significantly increased after the conditioning session. The level of fatigue sensation was shown not to be altered after the control session, i.e., non-conditioning session, previously [22]. In addition, characteristic ECDs in the PCC, with latencies of approximately 460 ms, could be specifically observed after the conditioning session. These results demonstrate that the classical conditioning of central inhibition can occur and that the PCC is involved in the neural substrates of central inhibition related to classical conditioning during physical fatigue.

Because the handgrip force of the right hand was decreased and the subjective level of the right hand for fatigue was increased after the handgrip trials (Fig. 2A), physical fatigue was induced by the conditioning session. Importantly, the subjective level of right hand fatigue after the second MEG session was increased after the conditioning session (Fig. 3A). In addition, sympathetic nerve activity during the MEG session assessed using ECG time-frequency analyses was increased after the conditioning session (Fig. 4B). Because increased sympathetic nerve activity has been observed by handgrip trials, i.e., physical fatigue [32], these observations demonstrate that physical fatigue could be classically conditioned.

Using metronome sounds as the conditioned stimuli and 60-min 2-back test trials as the unconditioned stimuli, our recent MEG study showed that the classical conditioning of mental fatigue sensation can occur and that the PCC (peak latency, 468.0±13.5 ms) was specifically activated after the conditioning, suggesting that this brain region is involved in the neural substrates of the mental fatigue sensation related to classical conditioning [22]. To support this finding, fear conditioning also caused increases in regional cerebral blood flow in the PCC, as assessed using H_2_^15^O positron emission tomography (PET) [33], [34]. As for the central inhibition, it was reported that, during a fatigue-inducing physical task, fatigue-related location shift of the movement-evoked MEG fields toward the PCC was caused and during the exhausting parts of the fatigue-inducing task and that the activation in the PCC preceded the activation in the primary sensory cortex, suggesting that the PCC is involved in the control of movement execution to preserve muscle efficiency and integrity, i.e., the central inhibition [35]. In addition, the PCC was shown to be a skeletomotor brain region [36] and this brain region was activated during pain-motor interactions [37]. These results support our suggestions that the classical conditioning of central inhibition can occur and that the PCC is involved in the neural substrates of central inhibition related to classical conditioning during physical fatigue. As for the insular cortex, it was difficult to determine whether this brain region is involved in the neural substrates of central inhibition during physical fatigue related to classical conditioning, because great levels of the auditory-evoked magnetic fields between 150 to 200 ms after the start of the metronome sounds occurred; thus it was hard to identify and isolate the ECDs in the insular cortex. Therefore, the roles of this brain region in central inhibition during physical fatigue and classical conditioning remain unclear.

There are three limitations to our study. First, we performed our study with a limited number of participants. In addition, while an absolute majority of patients is women, the participants in this study are all men. To generalize our results, studies involving a large number of participants including women ones are essential. Second, we did not assess neural activities during the conditioning session. It was difficult to evaluate neural activities during the fatigue-inducing handgrip trials by using MEG because of the electromagnetic noise caused by the handgrips. Thus, we adopted a study design to compare the neural activities before and after the conditioning session. Finally, it was difficult to assess the neural activities of the brain regions located deeply by using MEG. Therefore, some brain regions involved in the neural substrates of central inhibition related to classical conditioning (for example, the amygdala is reported to be related to the classical conditioning [38] as well as fatigue [39] and the orbitofrontal cortex is reported to be associated with fatigue sensation [40]) might be missed because of the limitations of MEG. Future studies using other neuroimaging techniques, such as fMRI and PET, would address this limitation.

In conclusion, we demonstrated that the classical conditioning of central inhibition can occur and that the PCC is involved in the neural substrates of central inhibition related to classical conditioning during physical fatigue. Despite its obvious importance, we still know little about central inhibition, and in particular, its contribution to the pathophysiology of chronic fatigue in human diseases or syndromes. Our findings may help to clarify the mechanisms of central fatigue related to classical conditioning as well as to develop treatment methods for patients suffering from chronic fatigue.
